# Supplementary material for: Targeted Co-Delivery of Gefitinib and Rapamycin by Aptamer-Modified Nanoparticles Overcomes EGFR-TKI Resistance in NSCLC via Promoting Autophagy
Source: Int J Mol Sci. 2022 Jul 21;23(14):8025. doi: 10.3390/ijms23148025 (PMC9316473; doi:10.3390/ijms23148025)
Supplement: Supplementary file 1 [file ijms-23-08025-s001.zip › ijms-1808368-supplementary.pdf]

A

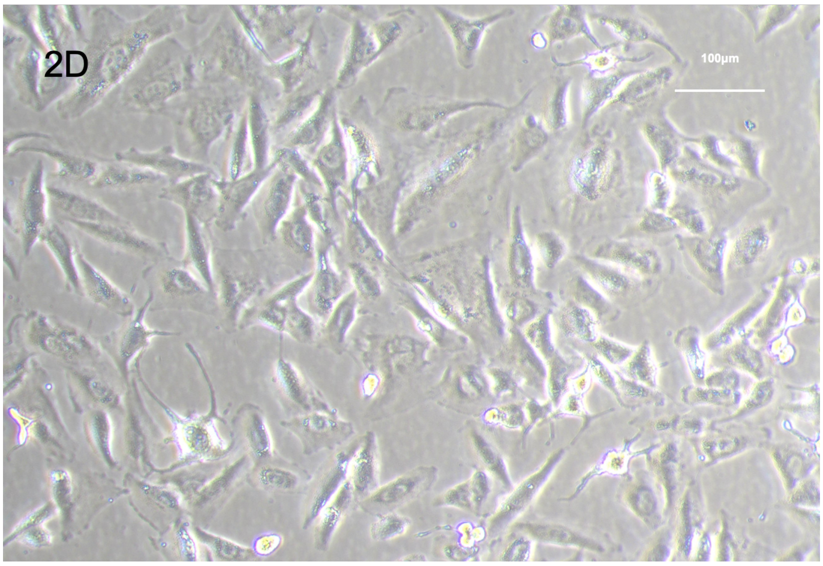

B

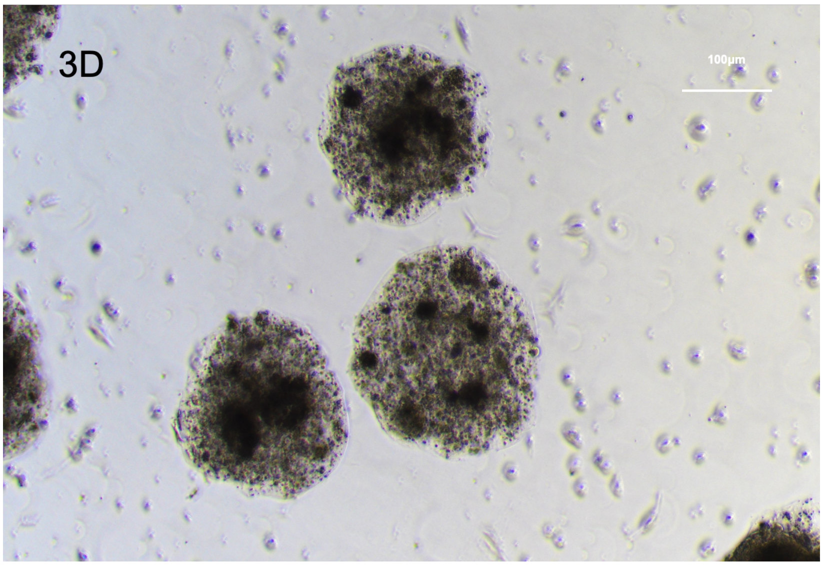

Supplementary Figure S1. Analysis of H1975 cell morphology. (A) 2D cultures. (B) 3D cultures.

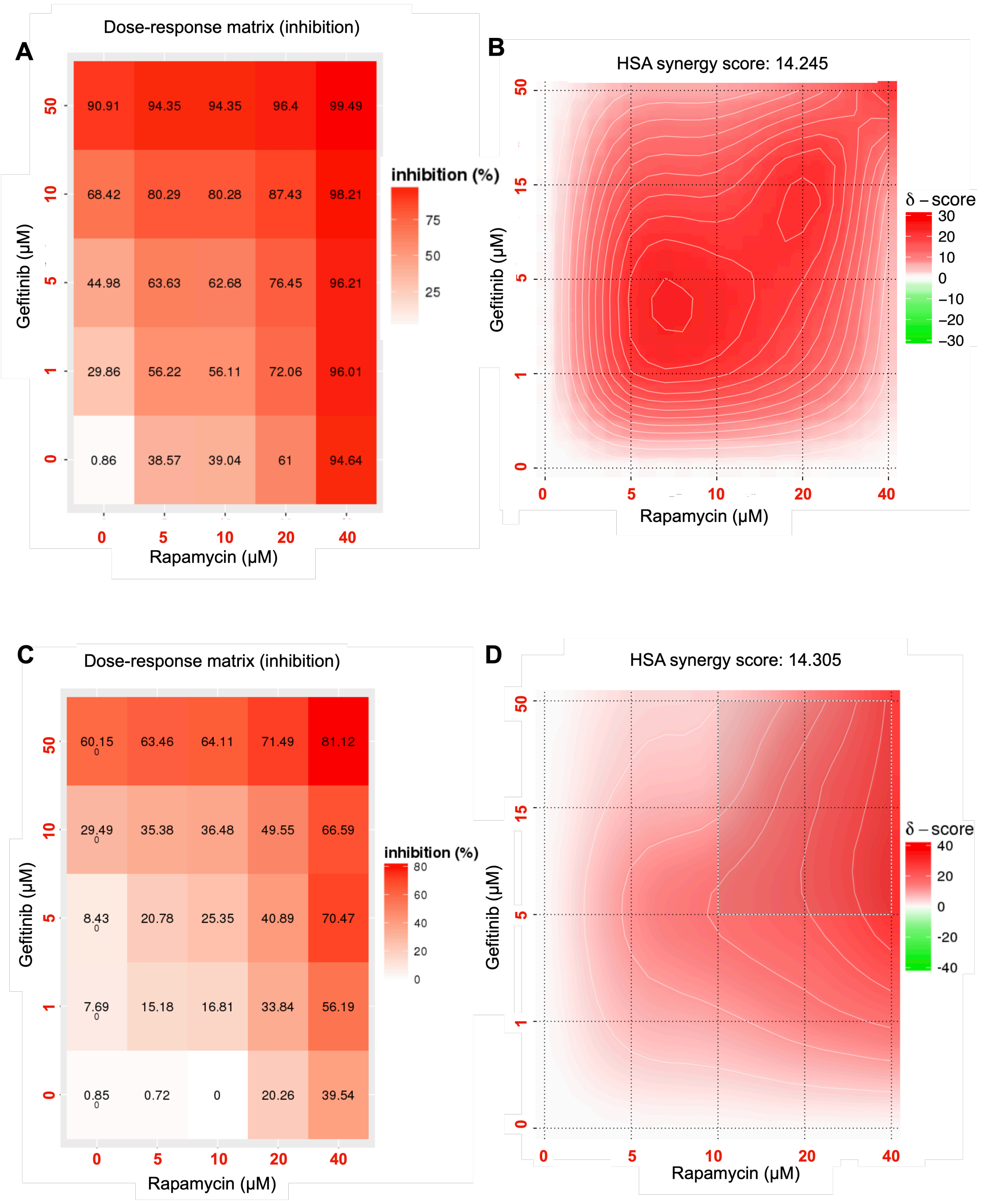

Supplementary Figure S 2. Dose response matrix and synergy score of gefitinib rapamycin combination treatment on H1975 cells. (A) Dose response matrix in 2D cultures. (B) Synergy score of gefitinib and rapamycin combination in 2D cultures. (C) Dose response matrix in 3D cultures. (D) Synergy score of gefitinib and rapamycin combination in 3D cultures.

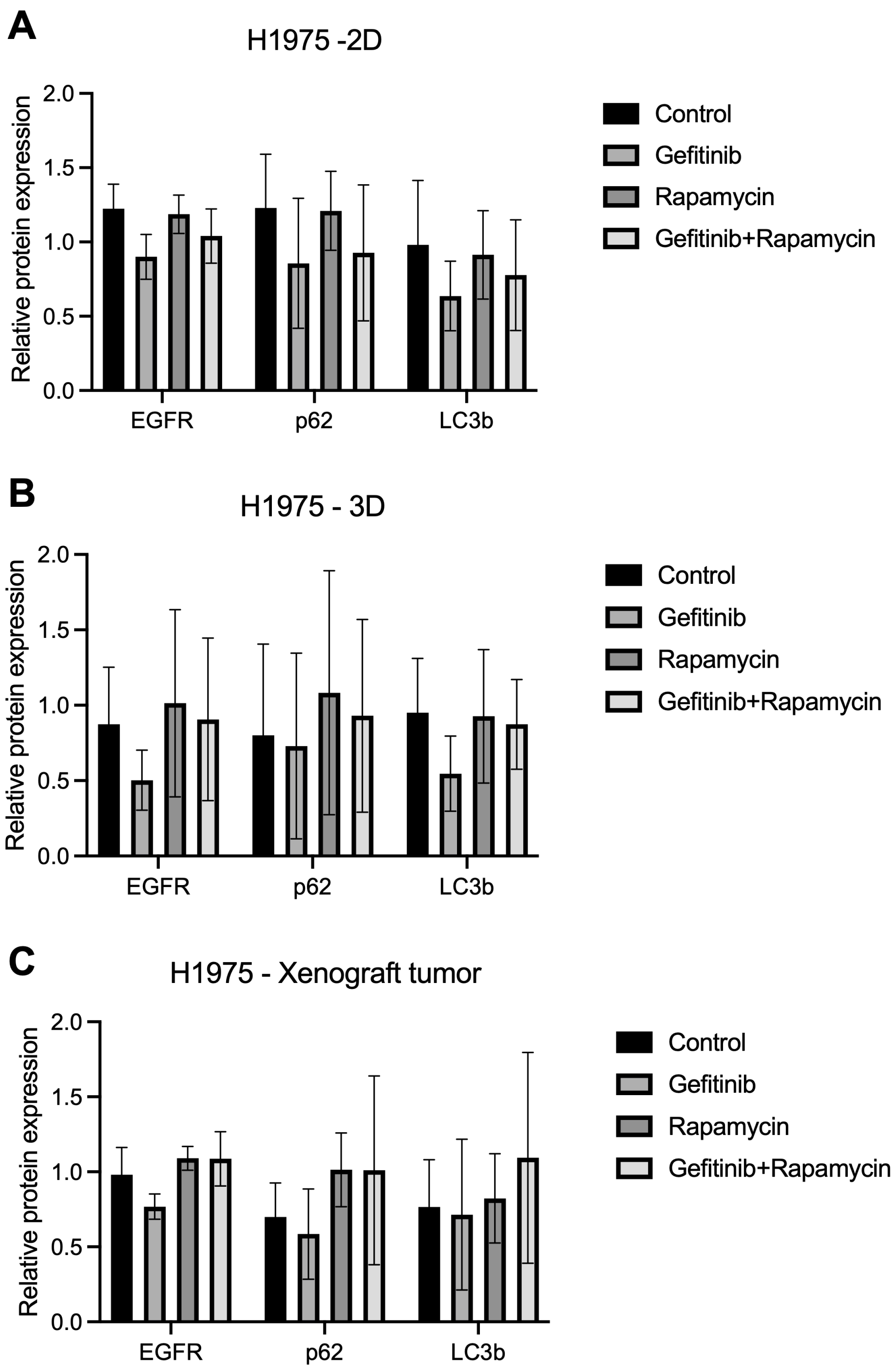

Supplementary Figure S3. Relative protein expressions in H1975 cells. (A) EGFR, p62, LC3b expression comparing to β-Actin in 2D cultures. (B) EGFR, p62, LC3b expression comparing to β-Actin in 3D cultures. (C) EGFR, p62, LC3b expression comparing to β-Actin in Xenograft tissue. (n=3)

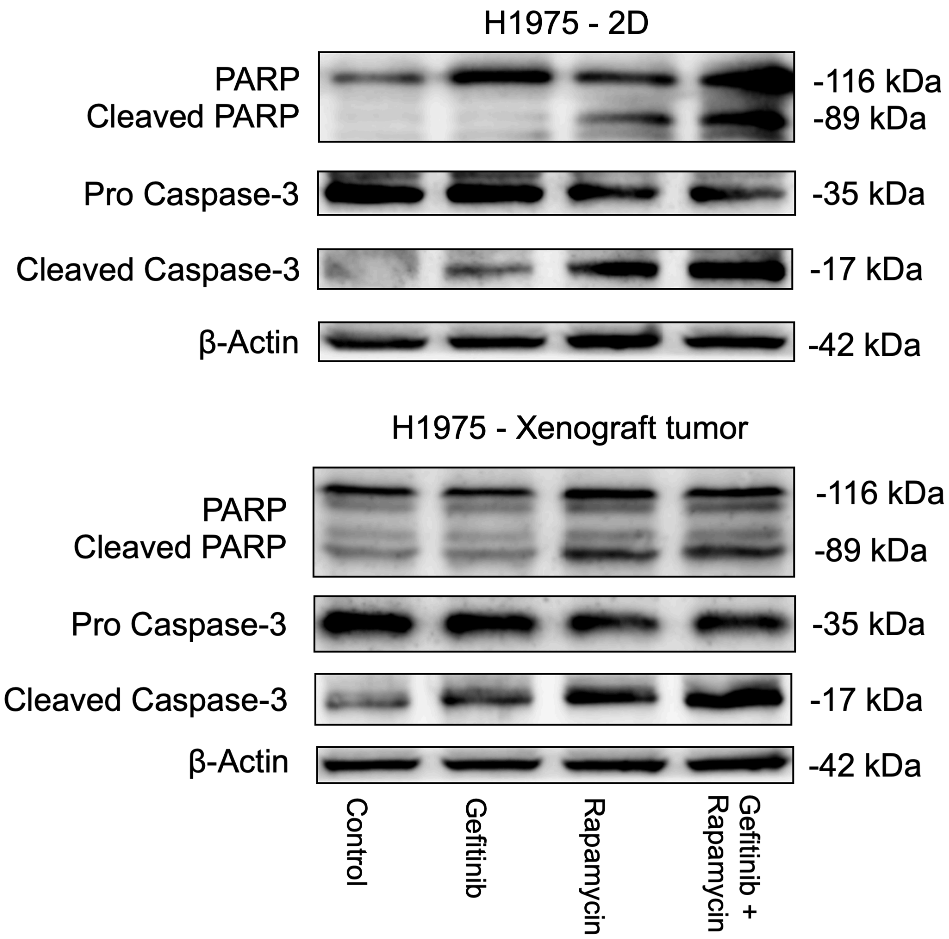

Supplementary Figure S4. Immunoblotting and quantities analysis of PARP, Caspase-3 and β-Actin protein expression in 2D cultures (gefitinib: 5 μM; rapamycin: 10 μM), and xenograft tumors (gefitinib: 150 mg/kg; rapamycin: 2 mg/kg).

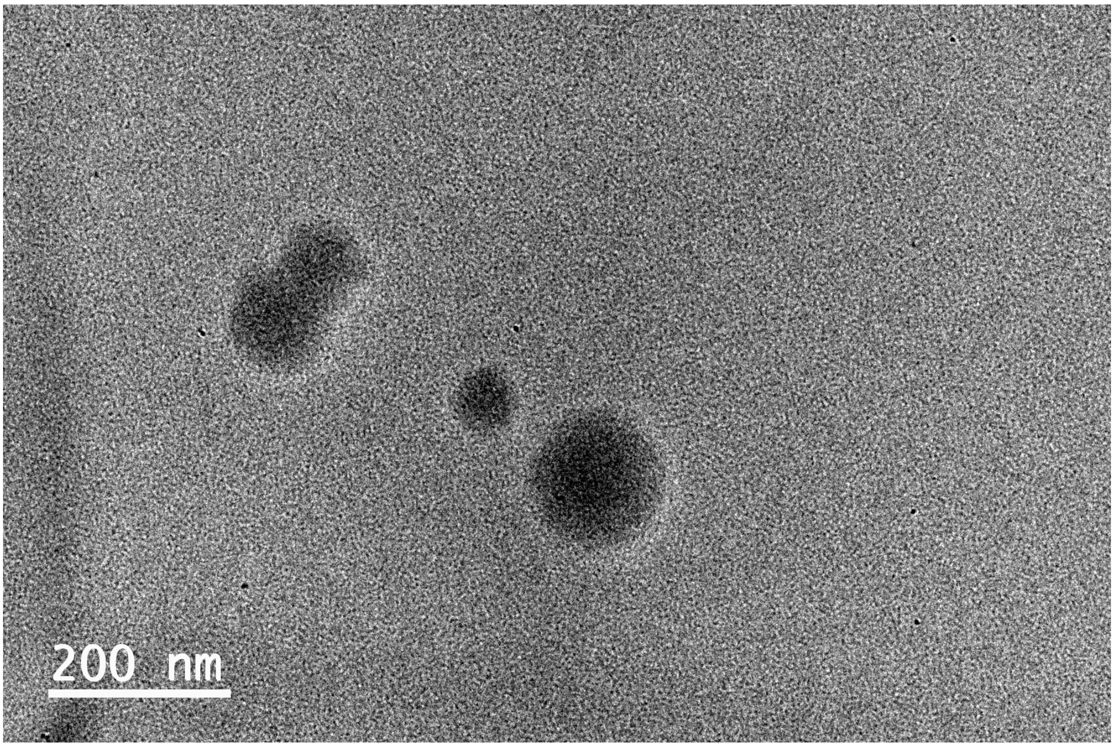

Supplementary Figure S5. TEM picture of NP.

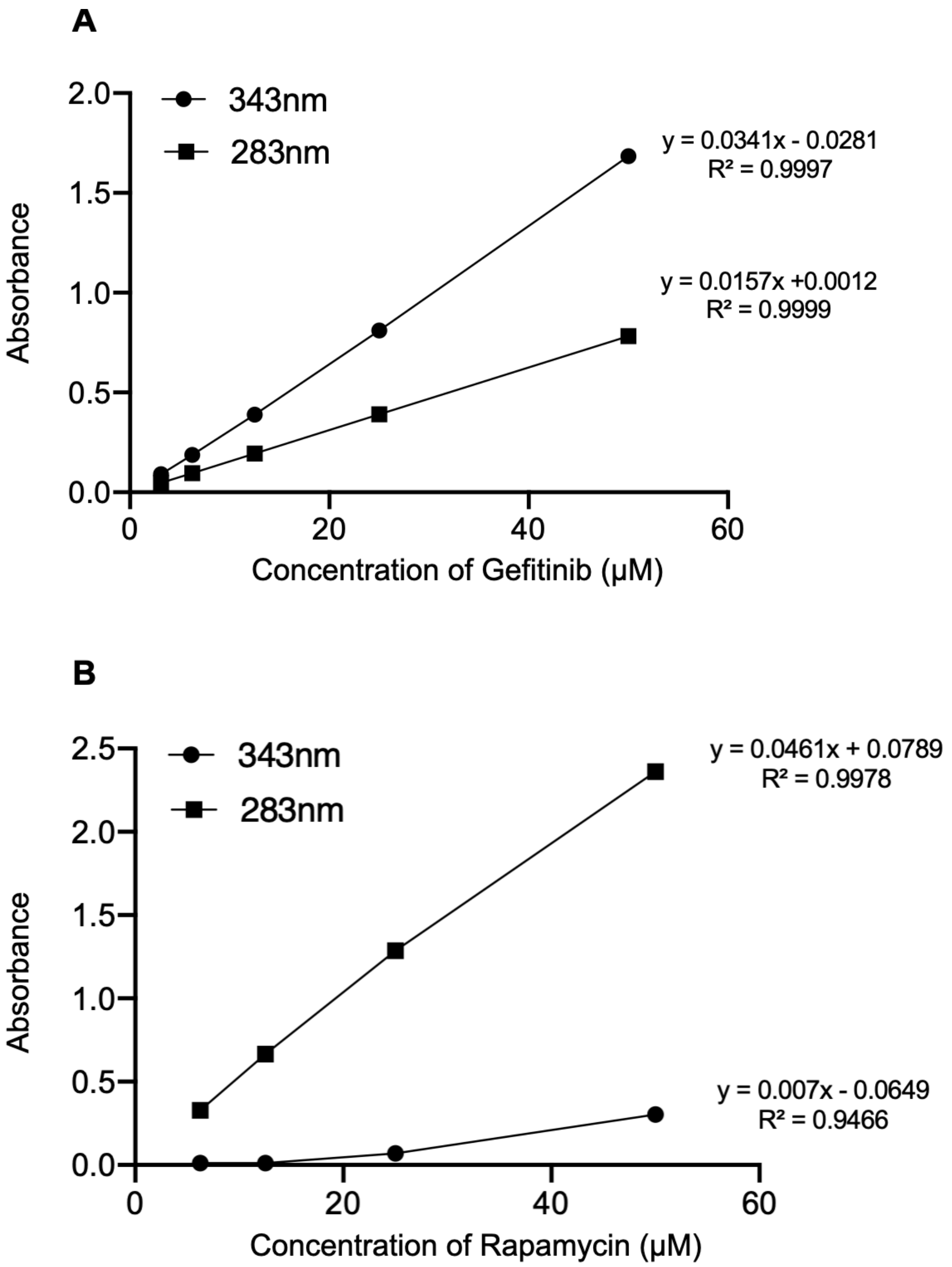

Supplementary Figure S6. The standard curve fit of gefitinib (A) and rapamycin (B) at 343 nm and 283 nm using a UV-Vis spectrophotometer.
